# Efficacy of palliative hemostatic radiotherapy for tumor bleeding and pain relief in locally advanced pelvic gynecological malignancies

**DOI:** 10.1007/s00066-024-02319-2

**Published:** 2024-11-12

**Authors:** Eva Meixner, Line Hoeltgen, Lisa A. Dinges, Semi Harrabi, Katharina Seidensaal, Fabian Weykamp, Philipp Hoegen-Sassmanshausen, Maria Vinsensia, Laila König, Maximilian Deng, Jürgen Debus, Juliane Hörner-Rieber

**Affiliations:** 1 Department of Radiation Oncology, Heidelberg University Hospital, Im Neuenheimer Feld 400, 69120 Heidelberg, Germany; 2 Heidelberg Institute of Radiation Oncology (HIRO), 69120 Heidelberg, Germany; 3 National Center for Tumor diseases (NCT), Heidelberg, Germany; 4 Heidelberg Ion Therapy Center (HIT), Im Neuenheimer Feld 450, 69120 Heidelberg, Germany; 5 Clinical Cooperation Unit Radiation Oncology, German Cancer Research Center (DKFZ), Im Neuenheimer Feld 280, 69120 Heidelberg, Germany; 6 Department of Radiation Oncology, University Hospital Düsseldorf, Düsseldorf, Germany

**Keywords:** Cancer vaginal bleeding, Transfusion, Hypofractionated radiation therapy, Anticoagulation, Re-irradiation

## Abstract

**Purpose:**

The appearance of symptomatic tumor-related vaginal bleeding and pain in advanced incurable cancer patients with pelvic gynecological malignancies remains a therapeutic challenge in oncological treatment. The aim of our analysis was to evaluate the efficacy and safety of palliative hemostatic radiotherapy.

**Methods:**

We retrospectively identified patients who had received palliative hemostatic radiotherapy (RT) at our institution between 2011 and 2023 and evaluated acute toxicity, local control, cessation of bleeding, and pain relief.

**Results:**

In total, 40 patients with a median planning target volume of 804 cm^3^ were treated with a median total dose of 39 Gy in 13 fractions, resulting in 6‑month and 1‑year local control rates of 66.9 and 60.8%, respectively. No higher-grade (>grade III) acute RT-induced toxicity appeared. Complete cessation of bleeding was achieved in 80.0% of all patients after a median of 16 days and pain relief was documented in 60.9% at first follow-up. 37.5% of the women required a blood transfusion and 25% an additional tamponade with local hemostatic agents. Successful stopping of bleeding was significantly less frequent in patients receiving anticoagulation concurrently with radiation and in the case of infield re-irradiation. Patients with a higher total RT dose had cessation of bleeding significantly more often, with a cut-off value of at least EQD2 (α/β = 10) = 36 Gy. The applied RT technique and planning target volume had no significant influence on the occurrence of bleeding cessation.

**Conclusion:**

Palliative hemostatic radiotherapy for locally advanced pelvic gynecological malignancies is safe and effective in achieving high control rates of hemostasis in tumor bleeding and pain relief.

## Introduction

With about 342,000, 207,000, and 97,000 cervical, ovarian, and endometrial cancer deaths worldwide in 2020, patients with locally advanced pelvic gynecological malignancies represent a substantial population of patients requiring palliative treatment in the end stages of their disease [1].

Cancer patients frequently suffer from symptoms like bleeding, pain, and infection resulting from local tumor growth or blood vessel infiltration and invasion along with tumor-driven angiogenesis [2]. In patients with advanced cancer stages of pelvic gynecological malignancies, clinical symptoms of bleeding occur in about 10% and treatment remains challenging [3]. Depending on the anatomic site and bleeding intensity, it can lead to a severe deterioration in quality of life and may require inpatient treatment, fluid replacement, and blood transfusion. Advanced cancer stages are often accompanied by vein thrombosis or acute pulmonary embolism requiring anticoagulation therapy, which makes bleeding even more likely.

Therapeutic options include invasive percutaneous or endoscopic interventions with embolization, coagulation, and surgical intervention or noninvasive approaches with compression, application of antifibrinolytic/hemostatic agents, or hemostatic radiotherapy (RT) [3, 4].

For managing bleeding in advanced cancer stages, considerations regarding the extent of therapy and supportive measures as well as patients’ preferences and life expectancy have to be made. In this context, treatment-related severe morbidity after pelvic exenteration for gynecological tumors has been shown to remain above 50% [4]. The choice of treatment must take into account the patient’s condition, comorbidities, and previous oncological therapies, and the goal of improving quality of life and symptoms must be weighed against a strong burden-to-benefit ratio.

Radiotherapy is available and widely used for oncological treatments, and small series have described radiotherapy to be effective for hemostatic palliative care, although dose-fractionation regimes and treatment techniques vary substantially [5–7]. External-beam RT has been reported to reduce hemoptysis and improve quality of life in patients with non-small cell lung cancer [8]. However, for patients with primary genitourinary, gynecologic, and gastrointestinal cancers, data are limited regarding the optimal approach, efficacy, and clinical benefit of RT in controlling bleeding and symptoms [7]. As such, we aimed to analyze clinical outcomes and influencing factors in women treated with palliative RT for advanced pelvic gynecological cancers.

## Materials and methods

In this single institutional retrospective analysis, we reviewed all patients who were treated between 2011 and 2023 with palliative pelvic RT for symptomatic locally advanced pelvic gynecological malignancies comprising cancers of the uterus, cervix, ovaries, vulva, and vagina. Ethics approval for the analysis and a waiver of written informed consent was granted by the local ethics review board (S-453/2021). The study was performed following the institutional guidelines, principles of good clinical practice, and the Declaration of Helsinki of 1975 in its most recent version.

### Patient and treatment data

Patient demographics; tumor- and radiation-specific data; and toxicity, radiology, and oncological outcomes were reviewed. Symptoms at the beginning of, during, and after RT, including bleeding (present or not present) and pain, as well as toxicities were identified. Acute (≤ 90 days) and late (> 90 days) toxicity was graded according to the Common Terminology Criteria for Adverse Events (CTCAE, version 5.0). Accordingly, the grading scheme for general pain was used and could refer to any anatomic region (e.g., abdominal, gastrointestinal, rectal, …). It was classified by the treating radiation oncologist as “mild” (CTCAE grade I), “moderate” (CTCAE grade II), or “severe” (CTCAE grade III).

All patients received RT in a palliative setting with external-beam RT (EBRT). Target volume delineation was performed on computed tomography planning scans with a slice thickness of 3 mm. The clinical target volume included the primary or recurrent tumor lesion with or without regional lymph nodes depending on tumor extension. Treatment technique, total dose, and fractionation as well as margins of 5–7 mm for planning target volumes (PTV) were determined by the responsible radiation oncologist. The dose constraints to surrounding organs-at-risk were in accordance with common recommendations [9, 10]. EBRT was delivered with a 6-MV linear accelerator (Siemens Mevatron, Erlangen, Germany or Elekta Versa, Stockholm, Sweden) either as three-dimensional conformal RT (3D-CRT) or intensity-modulated radiotherapy (IMRT). The equivalent total doses in 2‑Gy fractions (EQD2) were calculated with an assumed α/β ratio of 10 Gy for tumor tissue.

### Oncological follow-up

Clinical visits with clinical and oncological data and toxicities and radiological imaging results were reviewed for each patient during RT and follow-up. Overall survival (OS) was defined as the time from the end of RT until last contact or date of death. Local (LC) and distant control (DC) were defined from the end of RT until local progression of the primary tumor and progression or development of new distant metastases, respectively.

### Statistical analysis

Kaplan–Meier analyses were used to calculate the oncological outcome rates, and the log-rank test or Cox regression analysis was used to further compare subgroups, using a *p*-value of less than 0.05 as statistically significant. To assess the influence of prognostic cofactors, uni- and multivariate Cox proportional hazards ratios with a 95% confidence interval were applied. Data were compared using Mann–Whitney U or Pearson chi-square tests for continuous and categorical data, respectively. Statistical analysis was performed with IBM SPSS statistical software (version 28; IBM Corp., Armonk, NY, USA).

## Results

### Patient and treatment characteristics

Between May 2011 and September 2023, 46 patients were scheduled to receive palliative pelvic RT. Six patients were excluded from the analysis due to a switch to a curative concept (*n* = 3), refusal of RT (*n* = 2), and death before start of treatment (*n* = 1). The outcomes for a total of 40 patients with a median age of 69 years and a median follow-up time of 9.7 months (range 0.1–73.1) were analyzed.

The most common primary tumor was endometrial cancer (*n* = 16; 40.0%), followed by cervical cancer (*n* = 10; 25.0%), vulvar cancer (*n* = 8; 22.2%), and ovarian cancer (*n* = 6; 15.0%).

Previous therapy included oncological surgery in 25 patients with a median time of 20 months (range 2–134 months) prior to RT, including a hysterectomy in 16 women. No patient received simultaneous systemic therapy during RT.

Prior RT was documented in 8 patients with a median time interval of 17 months (range 4–223 months) and a median RT dose of 54 Gy (range 37–104 Gy; EQD2 α/β = 3; *n* = 6 EBRT, *n* = 2 vaginal brachytherapy, and *n* = 1 EBRT and endocervical brachytherapy).

**Fig. 1:**
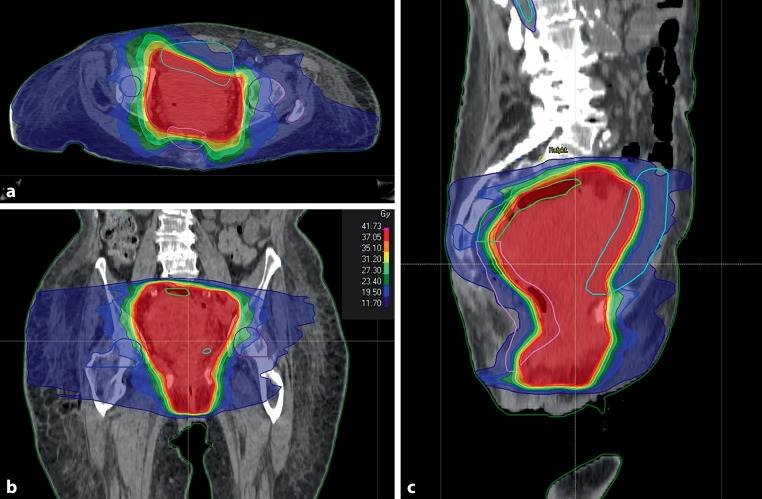
An 83-year-old woman with palliative hemostatic RT for endometrial cancer receiving 6‑MV photon intensity-modulated radiotherapy with 39 Gy in 13 fractions for a planning target volume of 1634 cm^3^ resulting in cessation of bleeding after 12 days (equivalent dose in 2‑Gy fractions for α/β of 10 = 24 Gy) (**a**: axial, **b**: coronar and **c**: sagittal computed tomography slices with the resulting radiotherapy plan)

The median Karnofsky performance score before the start of RT was 70% (range 50–90%). The median time from the first consultation at our department to the start of RT was 8 days (range 0–177). To a median pelvic PTV of 804 cm^3^ (range 82–3814 cm^3^), a median total dose of 39 Gy (range 20–45 Gy) in a single dose of 3 Gy (range 3–4 Gy) in 13 fractions (range 5–15) was applied. For 5 patients with vulvar cancer the PTV additionally included the inguinal lymph nodes. Figure 1 shows an example of a palliative hemostatic pelvic IMRT treatment.

Radiotherapy treatment included IMRT for 28 (70.0%) and 3D-CRT techniques for 12 patients (30.0%). Premature termination of RT was present in 4 (10.0%) patients in favor of best supportive care. Detailed patient and treatment characteristics with dose fractionation regimes of all patients are presented in Tables 1 and 2**.**

**Table 1:** Patient and treatment characteristics

Characteristic	Value/median (range or percentage)
*Median age at RT*; years	69 (40–94)
*Primary cancer*	
Endometrial cancer	16 (40.0%)
Cervical cancer	10 (25.0%)
Vulvar cancer	8 (20.0%)
Ovarian cancer	6 (15.0%)
*Oncological status at start of RT*	
Localized	10 (25.0%)
Metastasized	30 (75.0%)
*Time from first diagnosis to start of palliative RT*; months	11.8 (0.7–224.0)
*Total EQD2 (α/β* *=* *10) dose;* Gy	42 (13–49)
*Total EQD2 (α/β* *=* *10) dose until stop of bleeding*; Gy	39 (3–49)
*Total EQD2 (α/β* *=* *3) dose until stop of bleeding*; Gy	43 (4–54)
*Application of systemic therapy*	
Before RT	20 (50.0%)
Simultaneous to RT	0
After RT	20 (50.0%)

*EQD2* equivalent dose in 2 Gy fractions; *RT* radiotherapy

**Table 2:** Dose fractionation regimes used

Total dose [Gy]	Dose per fraction [Gy]	Fractions	EQD2 [Gy] (α/β = 10)	BED [Gy] (α/β = 10)	Number of patients
20	4	5	23.3	28	2
21	3	7	22.8	27.3	1
30	3	10	32.5	39	4
36	3	12	39.0	46.8	7
39	3	13	42.3	50.7	20
42	3	14	45.5	54.6	1
45	3	15	48.8	58.5	5

*BED* biologically effective dose. *EQD2* equivalent dose in 2‑Gy fractions (α/β = 10)

### Symptomatic outcome and influencing factors

Inpatient treatment during RT was necessary in 77.5% of the women. Fifteen (37.5%) patients received a transfusion of red blood cells (range 2–12) during RT, and 10 (25.0%) patients required additional application of vaginal tamponade with local hemostatic agents. Complete cessation of bleeding was achieved in 80.0% (*n* = 32) of all patients after a median time of 16 days (range 1–78 days) and a median EQD2 (α/β = 10) dose of 39 Gy (range 3–49 Gy). Patients treated with a higher total RT dose had cessation of bleeding significantly more often (*p* < 0.0001), with a cut-off value of at least EQD2 (α/β = 10) = 36 Gy.

Before the start of RT, 9 patients (22.5%) suffered from a symptomatic vein thrombosis or acute pulmonary embolism requiring therapeutic anticoagulation, and 5 patients received prophylactic anticoagulation. Successful cessation of bleeding was significantly less frequent in patients receiving anticoagulation concurrently with radiation (*p* < 0.0001) and in patients with infield re-irradiation concepts (*p* = 0.018). The applied RT technique (*p* = 0.168), PTV volume (*p* = 0.973), tumor entity (*p* = 0.252), and additional application of vaginal tamponade with local hemostatic agents (*p* = 0.361) had no significant influence on the occurrence of bleeding cessation.

Twenty-three (57.5%) patients suffered from pain (grade I: *n* = 12, grade II: *n* = 5, grade III: *n* = 6) before the start of RT. At the first follow-up after palliative RT, relief from pain was documented in 14 (60.9%), one woman presented with a deterioration of pain symptoms (*n* = 1, 4.3%), and the remaining patients remained stable.

### Toxicity and oncological outcome

Acute RT-induced toxicity included only low-grade gastrointestinal symptoms in 42.5% of patients (grade I *n* = 12, grade II *n* = 5) and genitourinary problems in 30.0% (grade I *n* = 11, grade II *n* = 6). No higher-grade RT-induced toxicity occurred. No significant correlation between the applied dose and toxicity could be found. Of note, 3 patients reported gastrointestinal symptoms (grade III *n* = 3, grade IV *n* = 1) prior to RT, with a deterioration of pre-existing symptoms with abdominal infection exacerbation in five women (grade III *n* = 3, grade IV *n* = 2) during RT.

At the end of the observation period, 17 (42.5%) patients were still alive. The 6‑month, 1‑year, and 2‑year OS rates were 66.9%, 60.8%, and 30.0%, respectively. Local failures were detected in 12 women (30.0%) after a median time to relapse of 3.9 months (range 1.4–14.5), resulting in 6‑month, 1‑year, and 2‑year LC rates of 66.9%, 60.8%, and 57.7%, respectively. The LC was significantly superior in patients who received a total applied EQD2 dose of at least 36 Gy (*p* = 0.011).

Chest and abdominal imaging were performed for distant staging purposes for a subgroup of 30 patients, resulting in 6‑month, 1‑year, and 2‑year DC rates of 47.6%, 37.0%, and 26.4%, respectively. The most common progressive metastases were in the extrapelvic lymph nodes (*n* = 8), liver (*n* = 7), peritoneum (*n* = 6), lung (*n* = 2), bone (*n* = 2), soft tissue (*n* = 2), and spleen (*n* = 1).

## Discussion

Our study consisted of a large group of palliative patients with locally advanced gynecological malignancies and demonstrated an effective response to hemostatic RT, with relief from symptomatic bleeding and pain with a moderately hypofractionated RT regimen.

Radiotherapeutic treatment represents a widely available noninvasive method for the treatment of palliative cancer patients. Hypofractionated regimens for palliative RT in the setting of painful bone metastases and relief from hemoptysis for lung or gastric cancer patients or superior vena cava syndrome are well described and have shown a high rate of symptom relief in palliative treatment [11–15].

Historically, patients with palliative pelvic malignancies of gynecologic, bowel, and prostate cancers were treated in a prospective non-randomized RTOG study by Spanos et al. with a schedule of 10 Gy in one fraction with once-monthly sessions. After a total of three fractions, complication rates were so high for gastrointestinal side effects that a protocol switch to a single dose of 3.7 Gy per fraction was performed [16, 17]. Halle et al. also applied 10-Gy fraction RT for palliative endometrial and cervical cancer patients, with bleeding cessation rates of 60%; however, there were serious treatment-related complications in 12% of patients [18]. Current RT protocols deliver more moderate hypofractionation regimens of 20–40 Gy in 5–20 fractions with minimal toxicity and effective palliative symptom relief, although the optimal treatment regimen remains unclear [19, 20]. While the main objective of our palliative RT approach—and in palliative care in general is the alleviation of symptoms, treatment-associated toxicity should be reduced to a minimum. With a median dose concept of 39 Gy in 13 once-daily fractions, no higher-grade (≥ 3) toxicity was observed in our study.

Butala et al. compared short-course RT (≤ 5 fractions, > 3.5 Gy per fraction) to conventionally fractionated palliative RT (> 5 fractions) in 33 patients with bleeding gynecological malignancies of mostly uterine (42.4%) and cervical (30.3%) cancer [21]. They found grade III gastrointestinal toxicity in 9.1% and bleeding control in 90.9% of the patients after a median time to hemostasis response of 13 days. Interestingly, no difference between the symptom response rates of the two fractionation schemes could be found. Caravatta et al. delivered short-course accelerated RT up to 18 Gy in 4.5-Gy bidaily fractions with only grade I and II toxicity to pelvic malignancies and achieved pain relief in 91% of patients [22].

Further, successful cessation of bleeding within 10 days from the start of RT was documented in 88.8% of patients in a study by Macchia et al. comprising 9 endometrial cancer patients [23]. Patients received 10 fractions of 3 Gy and additional progestin-releasing intrauterine device insertion. With a similar dose concept, the patients in our study achieved comparable results, with 80% achieving cessation of bleeding with a clear dose–response effect with a cut-off EQD2 (α/β = 10) value of 36 Gy (BED_α/β_ _=_ _10_ = 46.8 Gy). Other studies reported a BED_α/β_ _=_ _10_ of ≥ 36 Gy as a significant factor for improved symptom control in the treatment of pelvic malignancies of different tumor entities [24].

In contrast to this, the study of Kombathula et al. could not find a dose–response correlation with symptom control in the palliative pelvic RT of 184 women with gynecological tumors and thus supported the use of shorter low-dose RT regimens [25]. Overall, data and research on RT for gynecological palliative concepts are limited and inconsistent, and the optimal dosage and fractionation concepts remain unclear. Table 3 summarizes the discussed studies on hemostatic and palliative pelvic RT.

**Table 3:** Selected studies on pelvic hemostatic and palliative radiotherapy

Author	Title	Number of patients	RT fractionation schedules	Bleeding and pain control	Side effects
Rasool et al. [5]	Hypofractionated radiotherapy as local hemostatic agent in advanced cancer	*n* = 5 gynecological (overall *n* = 25)	20 Gy in 5 fractions or 15 Gy in 5 fractions using cobalt 60	88% (22/25 patients) complete cessation (equal efficacy of 15 and 20 Gy)	No higher-grade toxicity
Aoshika et al. [6]	Safety and efficacy of palliative radiotherapy (25 Gy × 5 fractions) for symptomatic pelvic tumors	*n* = 14 gynecological (overall *n* = 34)	25 Gy in 5 fractions	82% (14/17 patients) hemostatic response 78% (14/18 patients) pain relief	Acute diarrhea grade I (*n* = 3), Acute dermatitis grade I (*n* = 1)
					Acute urinary frequency grade I (*n* = 1)
					Late AEs have not been observed
Spanos et al. [16]	Late effect of multiple daily fractions palliation schedule for advanced pelvic malignancies (RTOG 8502)	*n* = 290/surviving 90 days *n* = 193, 40% gynecological	44.4 Gy in 12 fractions (twice a day) with a rest 3–6 or 2–4 weeks after 14.8 Gy and 29.6 Gy	n. a., only oncological outcome reported	Crude late complications rate: 6%
					Cumulative incidence: 6.9% by 18 months
Spanos et al. [17]	Palliation of advanced pelvic malignant disease with large-fraction pelvic radiation and misonidazole: final report of RTOG phase I/II study	*n* = 20 gynecological (overall *n* = 46)	30 Gy in 3 fractions at 4‑weeks intervals (+misonidazole)	n. a., only oncological outcome reported (43% objective response)	11% grade III and 19% grade IV GI toxicities leading to a protocol replacement
Halle et al. [18]	1000 cGy single-dose palliation for advanced carcinoma of the cervix or endometrium	*n* = 42	10 Gy in 1 fraction, repeated once or twice at monthly intervals as necessary	60% cessation of bleeding 22% pain relief (permanent in approximately half of the patients)	11.9% (5/42 patients) serious treatment complications (4 occurred more than 10 months after treatment)
Carrascosa et al. [20]	Palliation of pelvic and head and neck cancer with paclitaxel and a novel radiotherapy regimen	*n* = 20 (pelvic and head and neck)	3.7 Gy twice a day for 2 days every 3 weeks for three cycles, in total: 44.4 Gy in 12 fractions (+paclitaxel)	89.5% (17/19 patients) palliation of their presenting symptoms, 42% (8/19 patients) effective benefit lasting more than 6 months	No late toxicities have been observed
Butala et al. [21]	A retrospective study of rapid symptom response in bleeding gynecologic malignancies with short-course palliative radiation therapy: less is more	*n* = 33	Median BED 37.5 Gy (total dose range: 8–50.4 Gy in 1–28 fractions) Short-course RT in 54.5% (18/33) of patients: median BED 28.0 Gy, 8–25 Gy in 1–5 fractions	78.8% (26/33 patients) hemostatic response during RT Median time to initial and maximal response: 5 and 13 days (no significant difference to conventional fractionation) Average durability: 5.4 months Re-bleeding rate 9.9%	9.9% (3/33 patients) grade ≥ III GI toxicity
Caravatta et al. [22]	Short-course accelerated radiotherapy in palliative treatment of advanced pelvic malignancies: a phase I study	*n* = 27 (48% gynecological)	Three dose escalation steps: 14 Gy in 4 fractions 16 Gy in 4 fractions 18 Gy in 4 fractions (twice daily)	41.7% (5/27 patients) complete pain relief	Only grade I–II acute toxicities
Macchia et al. [23]	Progestin-releasing intrauterine device insertion plus palliative radiotherapy in frail, elderly uterine cancer patients unfit for radical treatment	*n* =9	30 Gy in 10 fractions (+progestin-releasing intrauterine device)	88.8% (8/9 patients) complete bleeding cessation median duration: 18 months	No severe RT-related toxicity documented
Ogita et al. [24]	Palliative radiotherapy for gross hematuria in patients with advanced cancer	*n* = 2 gynecological (overall *n* = 53)	Most frequently used: 30 Gy in 10 fractions (26%), 20 Gy in 5 fractions (23%), 36 Gy in 12 fractions (21%)	76% gross hematuria free Median duration: 4.3 months	No grade ≥ III AE
Kombathula et al. [25]	Palliative radiotherapy in cancers of female genital tract: Outcomes and prognostic factors	*n* = 184	Most frequently used: 35 Gy in 15 fractions (33.6%), overall: 10–50 Gy in 1–20 fractions	77.7% and 21.1% complete and partial response of vaginal bleeding 56.6% and 25.3% complete and partial pain relief 80.4% subjective response in pre-RT symptoms	2.2% GI grade ≥ III, 3.8% urinary grade ≥ III, 2.7% other (vomiting, fatigue, pain) grade ≥ III

*AE* adverse events, *BED* biologically effective dose, *GI* gastrointestinal, *n.* *a.* not applicable, *RT* radiotherapy

Even though the chosen RT technique has been shown to result in lower toxicity with the use of advanced techniques such as IMRT compared to 3D-CRT or four-field boxes, no significant toxicity reduction could be found in our study with the use of IMRT [26, 27].

The main limitations of our study include its retrospective nature and the short follow-up time, although this is a reflection and consequence of the palliative setting. Due to the retrospective study design, systematic assessment with quality of life questionnaires was not possible, and symptom relief was categorized according to the clinical response. Similar to other studies, the median follow-up times in our population and the estimated 2‑year OS and LC rates of 30% and 57.7%, respectively, were low, and the palliative dosage did not aim to achieve long-term local control but rather palliative relief [25].

Further, only a small number of patients (*n* = 3) with short-course RT over five consecutive days were treated in our study. In palliative care, shorter treatment times may be useful in appropriate and well-selected patients. Even though our study suggested a dose–symptom response, no further evaluation of more intense hypofractionation concepts could statistically be performed.

As the main objective for a patient’s remaining lifetime is preservation of quality of life, treatments should minimize the efforts and need for transportation, as well as the number of treatment visits and fractions, and ensure effective relief of symptoms. However, there is still a lack of consensus in terms of the optimal palliative RT treatment schedule, and further research for defining tailored therapeutic concepts for palliative cancer patients is needed.

## Conclusion

Our clinical outcomes of hemostatic RT demonstrate its safety and effectiveness for palliative symptom relief in the treatment of women with advanced pelvic gynecological tumors, with high response rates for the control of tumor bleeding and pain relief. However, there is still a lack of consensus as to the optimal dosage, and fractionation regimes remain controversial.

## Data Availability

All data generated or analyzed during this study are included in this published article

## Abbreviations

3D-CRT: Three-dimensional conformal radiotherapy
BED: Biologically effective dose
DC: Distant control
EBRT: Externa-beam radiotherapy
EQD2: Equivalent total doses in 2‑Gy fractions
IMRT: Intensity-modulated radiotherapy
LC: Local control
OS: Overall survival
PTV: Planning target volume
RT: Radiotherapy
